# Part one: abuse liability of Vuse Solo (G2) electronic nicotine delivery system relative to combustible cigarettes and nicotine gum

**DOI:** 10.1038/s41598-022-26417-2

**Published:** 2022-12-21

**Authors:** Chris Campbell, Tao Jin, Elaine K. Round, Eckhardt Schmidt, Paul Nelson, Sarah Baxter

**Affiliations:** 1 RAI Services Company, 401 N. Main Street, Winston-Salem, NC 27101 USA; 2 Retired Employee of RAI Services Company, Winston-Salem, NC 27101 USA

**Keywords:** Randomized controlled trials, Addiction

## Abstract

Abuse liability (AL) of electronic nicotine delivery systems (ENDS) is relevant as the category increases in popularity as a potentially less-harmful alternative to cigarette smoking. AL assessments are important to the FDA in determining if a new product is appropriate for the protection of public health. This paper reports the results for Vuse Solo (G2 cartridge design) compared to high and low AL-comparators evaluated in an open-label, randomized crossover confinement AL study. The confinement design was adapted from previous ambulatory studies of Vuse Solo (G1 cartridge design) and included product familiarization sessions before each four-hour test session in which subjective measures, nicotine pharmacokinetics (PK), and physiological endpoints were assessed following a single 10-min ad libitum product use session. Product liking, intent to use again, suppression of urge to smoke, and nicotine PK were lower after use of Vuse Solo compared to cigarettes and higher after use of Vuse Solo compared to nicotine gum. No significant differences in blood pressure or heart rate were observed between the products pre- to post-product use. These data reinforce previous research and provide the scientific evidence to support regulatory decisions demonstrating that Vuse Solo has an AL profile lower than that of combustible cigarettes but higher than that of nicotine gum and, therefore, may be a suitable replacement for cigarette smoking for some adult smokers.

## Introduction

The abuse liability (AL) of a tobacco product has been defined as the likelihood that individuals will engage in persistent or problematic use and experience undesirable consequences as a result of use^1^. It is widely accepted that combustible cigarettes (CC) have the highest AL among tobacco- and nicotine-containing products^2–4^. One strategy for tobacco harm minimization is to ensure availability of less toxic but acceptably satisfying non-combusted alternatives; that is, products that can provide relief from cigarette craving and nicotine withdrawal effects with lower exposure to harmful or potentially harmful constituents^5,6^. Electronic nicotine delivery systems (ENDS) have been proposed as one such alternative^7^. An evaluation of the AL of ENDS compared to that of CC provides the FDA and the public health community with data to understand likelihood for long-term use of ENDS and subsequently their impact to both individual and population health^8,9^.

There are many physiological and psychological elements that contribute to a tobacco product’s AL. These include nicotine pharmacokinetics (PK), the ritual of using the product, perceived benefits, sensory aspects of the product, the balance between positive and negative subjective effects, and the ability to counteract feelings of withdrawal and craving^1,10–13^. Nicotine’s reinforcing effects are linked to the PK profile, such that products with slower or lower nicotine uptake are considered to be less reinforcing^14^. In addition, other attributes of tobacco product use contribute to the early experiences of craving relief, as demonstrated by studies with nicotine-free or low-nicotine products^15^. For example, nicotine-free e-liquids can perform on par with nicotine-containing e-liquids for endpoints related to cravings and subjective effects, especially when there are cognitive expectations of craving relief and psychological reward^13,16,17^. This is in contrast to other research which demonstrates that smokers can discriminate between products with and without nicotine and prefer products that contain nicotine^17–19^.

There is no single assessment that determines the relative AL of a tobacco product. Rather, conclusions about a products’ abuse liability are made by integrating the results of multiple types of assessments to predict a product’s potential to cause dependence^1^. These assessments can include clinical research studies to characterize nicotine PK after product use and concurrent evaluations of subjective measures such as product liking, reduction in urge to smoke, and cigarette cravings^20–24^. Behavioral economic demand analysis may also be used to assess the relative value of different product categories, where higher value is associated with higher AL^25,26^.

The Center for Tobacco Products within the US Food and Drug Administration (FDA CTP) considers AL evaluations integral to their determination of whether a new tobacco product is appropriate for the protection of the public health and thus eligible for marketing authorization. It is generally recognized that for potentially reduced risk tobacco products such as ENDS to be adopted by smokers as a replacement for CC, ENDS must be sufficiently satisfying and provide smokers with positive subjective experiences, but without encouraging use by non-smokers or smokers who otherwise would have quit smoking^5,27–30^. The FDA has not established an “acceptable” or “unacceptable” level of AL for new tobacco products, nor has it placed specific weight on different measures that factor into AL. However, the FDA noted that product liking and maximum effect are common primary outcome measures in ‘standard’ AL studies^8^. Further, comparisons to one or two other products with known levels of AL are needed to understand how the abuse liability of such a product compares to other relevant categories of tobacco products.

The present study design was based on FDA guidance regarding assessments of human abuse potential of drugs^31^ and the application of these methods to tobacco products^1,32^. As with standard AL evaluations of pharmaceutics, studies were performed with current experienced adult smokers to ensure study subjects were familiar with the effects of nicotine-containing products. Subjective measures of product liking were included as one of the primary endpoints with the timing of the subjective measures aligned with the nicotine pharmacokinetic assessments. Additional subjective, safety, and physiological measures were evaluated. A combustible cigarette was used as the positive control as it represents a nicotine product with a known, high AL. Nicotine gum was used as a reference control as it is generally accepted to be a product with low AL. There is a growing body of research using similar methods to study various types of ENDS^24,33,34^, including research on cig-a-like products such as Vuse Solo^22,23,35^.

Vuse Solo is a closed-system ENDS consisting of a power unit that works in combination with non-refillable e-liquid-containing cartridges. Vuse Solo was the first ENDS product to receive a marketing granted order from the FDA through the Premarket Tobacco Product Application (PMTA) process^36^. The authorization covered two versions of the Vuse Solo cartridge, designated as Generation 1 (G1) and Generation 2 (G2), both with Original (tobacco-flavored) e-liquids. Data supporting these applications included previously published clinical research with Vuse Solo G1 that demonstrated reduced abuse liability^22,23^, reduced environmental emissions^37^, and reduced biomarkers of exposure after short term use compared to continued use of CC^38^.

This paper is the first in a three-part series reporting on findings from several clinical studies of Vuse Solo G2. Part Two describes the PK of Vuse Solo G2 across four e-liquid flavors^39^. Part Three presents the results of a study to assess whether use of Vuse Solo G2 results in a reduction in exposure to harmful and potentially harmful constituents (HPHCs) after smokers are switched to the product for 5 days^40^. All studies reported in this three-part series were conducted to provide the FDA with evidence that Vuse Solo G2 is appropriate for the protection of the public health. They were included in the PMTAs submitted to the FDA for Vuse Solo G2.

This study contributes to the extant literature by demonstrating that the PK and subjective effects profiles for Vuse Solo G2 are consistent with prior findings for Vuse Solo G1. Furthermore, the FDA marketing order authorized the sale of Vuse Solo G2 (Original flavor) based on a population health standard. The results described in the current manuscript formed part of the basis for the FDA decision, making it of interest to the scientific and tobacco control community. Lastly, the protocol included product familiarization sessions and clinical confinement for the duration of the study, which were not part of prior research with Vuse Solo G1.

## Methods

### Study design and participants

This was a randomized, open-label, crossover study (ClinicalTrials.gov identifier: NCT03126357) designed to evaluate subjective effects, plasma nicotine uptake, and physiological measures following use of Original (tobacco) flavor Vuse Solo e-cigarettes with a second-generation (G2) cartridge design, relative to usual brand CC or nicotine gum. The crossover design was chosen to minimize variability and the number of subjects needed for evaluation. The study was completed at a single research center (Celerion, Lincoln, NE) and was reviewed and approved by Chesapeake Institutional Review Board (Columbia, MD). It was conducted in accordance with the ethical standards in the Declaration of Helsinki and applicable sections of the United States Code of Federal Regulations (21 CFR Parts 50, 54, 56 and 312 Subpart D), and ICH E6 Good Clinical Practice guidelines. The study included an abuse liability assessment of two additional vapor products using the same comparators, and these results will be discussed in a future publication.

Potential participants were recruited using standard advertising methods (print media, radio, and television) and from the study site’s existing recruitment database. Informed consent was obtained from all potential participants before initiation of any study events. Eligibility was assessed during a screening process. The eligible target population was male and female smokers aged 21–60 years determined by the principal investigator to be in reasonably good health. Eligible participants also self-reported smoking 10 or more non-mentholated filtered CC per day for at least the previous 6 months, typically smoked their first cigarette of the day within 30 min of waking, and reported not using any ENDS for the prior 30 days. Smoking status was confirmed by expired breath carbon monoxide measurements of ≥ 15 ppm. Women who were pregnant, breastfeeding, or aged ≥ 35 years and currently using systemic estrogen-containing contraception or hormone-replacement therapy were excluded from participation. All potential participants who expressed an interest in quitting smoking were excluded from the study and were encouraged to quit. After enrollment, subjects were randomized to product presentation sequences using a Williams Design to minimize assignment bias.

### Study products

Vuse Solo G2 Original is a vapor product comprising a rechargeable power unit (device) and a closed replacement cartridge containing approximately 0.5 ml of tobacco flavored e-liquid (RJR Vapor Company, Winston-Salem, North Carolina). The G2 cartridges employed in the present study are substantially similar to the product previously reported in Stiles et al. (G1 cartridges)^22^. The materials and construction of the two cartridges are very similar, with the primary differences being the outer tube material and the configuration of electronic components inside the cartridge. Although G1 and G2 cartridges are distinct products, they are equivalent in function, use, and aerosol delivery. The contain the same e-liquid formulations, which is a mix of propylene glycol (PG), glycerin (PG:glycerin ratio of 21:79), nicotine, water, and flavoring ingredients. The Vuse Solo Original e-liquid includes nicotine at 4.8% nicotine by weight (~ 57 mg/ml) and contains nicotine salts. The products used in the study were commercially available in the USA at the time the study was conducted.

The high- and low-AL comparators (usual brand cigarettes and Nicorette White Ice Mint nicotine polacrilex gum 4 mg [GlaxoSmithKline], respectively) were chosen in accordance with the 2017 FDA Guidance on Assessment of Abuse Potential of Drugs which discusses the use of positive and negative controls when designing studies^31^. Vuse Solo and nicotine gum were provided to the participants free of charge, whereas they provided their own usual brand cigarettes.

### Study procedures

The 11-day confinement period was divided into five different 48-h Study Periods which each lasted from mid-day on Study Period Day 1 until the end of the PK test session at mid-day on Study Period Day 3 (Supplementary Figure S1). After a period of a day and half of product familiarization with a minimum of six ten-minute uses of the Study Period assigned product, subjects participated in 4-h test sessions in which they used the assigned product ad libitum for 10 min (CC or ENDS) or 30 min (NRT) and subjective measures and blood samples for PK assessment were collected prior to, during, and for 240 min following the start of product use. Use parameters during the Trial Use Sessions aligned with those utilized in the test sessions. The test sessions occurred on Day 3 of each Study Period.

Product use parameters for ENDS and gum during the Trial Use Sessions and test sessions were as follows: ad libitum use of ENDS for approximately 10 min or one piece of nicotine gum for approximately 30 min of use according to the package insert (i.e., “chew and park” method). During the CC test session, a single CC was smoked to completion. Subjects could request additional trial use of non-CC assigned study product during the one and half days of Study Period prior to the abstinence period and subsequent test sessions. They could request their usual brand CC for ad libitum use throughout the study except during scheduled use of other products (i.e., product familiarization periods), for one hour prior to scheduled nicotine gum use, and during the abstinence periods. Subjects abstained from all nicotine-containing product use for at least 12 h overnight prior to test sessions to minimize the impact that residual nicotine might have on subjective effects. A new study period began upon completion of the test session of the preceding study period.

Test sessions were held on the morning of each Study Period Day 3 and involved product use as described above and subjective measures assessments, blood sampling for nicotine uptake, and physiologic measurements. Use of the three types of products during the test sessions occurred in separate areas of the clinic to avoid potential sensory cues that may have impacted responses to the subjective measures. Vuse Solo products were provided to the subjects with fully charged batteries, and the cartridges were weighed before and after use to determine the amount of e-liquid aerosolized.

### Study assessments

Five different subjective effects questionnaires were administered during the study on paper forms. Although not psychometrically validated, the subjective measures questionnaires were adapted from questions used for abuse liability assessment of psychoactive drugs. Similar versions of these questionnaires were used in the previously published AL studies of Vuse Solo^22,23^. Each questionnaire was provided to subjects on the day of study check-in for training purposes. The product liking (“At this moment, how much do you like the product?”) and product effects (“Rate the degree to which you feel positive/negative effects of the product at this moment”) questionnaires were completed at 15, 30, 45, 60, 90, 120, 150, 180 and 240 min following the start of product use during each test session. The urge to smoke (“How strong is your current urge to smoke your usual brand cigarette?”) questionnaire was completed prior to and at 5, 15, 30, 45, 60, 90, 120, 150, 180 and 240 min following the start of product use during each test session. The overall product liking (“Overall, how much do you like the product?”), and overall intent to use again (“Rate the degree to which you would like to use the product again”) questionnaires were completed only at the 240-min time point. All questionnaires were administered as 11-point numeric rating scales (NRS) of 0 to 10, with “strongly like/dislike,” “no urge,” “no positive/negative effects,” or “not at all” on the left anchor (“0”) and “strongly like/dislike,” “extremely strong urge,” “extremely positive/negative effects,” or “very much” on the right anchor (“10”). The NRS scale was used based on internal research indicating that the NRS scale is better understood by research subjects than the 100-point visual analog scale customarily used for measures of product liking. A midpoint descriptor “Neither like nor dislike” was included for the product liking and overall product liking questionnaires.

Venous blood samples for plasma nicotine concentration measurement were collected at − 5, − 0.5, 5, 7.5, 10, 15, 20, 30, 45, 60, 75, 90, 120, 150, 180 and 240 min relative to the start of product use during each test session with the − 0.5-min sample used as the preferred baseline sample. Samples were analyzed and nicotine concentrations determined in plasma using a validated liquid chromatography tandem mass spectrometry method at Celerion Global Bioanalytical Services (Lincoln, NE).

Blood pressure and heart rate were measured prior to product administration and at 15, 30, 45, 60, 120, 180 and 240 min following the start time of product use during each test session. Where the measurements coincided with blood sampling, the blood pressure and heart rate were taken after blood sampling to minimize the impact on nicotine PK results.

Safety and tolerability were evaluated based on data collected from physical and oral examinations, clinical laboratory tests, electrocardiograms, and adverse events (AEs). AEs were categorized according to an individuals’ assigned IP during each 48-h Study Period.

### Statistical analysis

Based on data from a previous study of the similar first-generation product^22^, the minimum number of subjects needed to complete all test sessions was 30 to allow for 80% power to detect the hypothesized 20% differences between Vuse Solo and CC with the significance threshold set at p = 0.0013 level (Bonferroni-adjusted for multiple comparisons) for the primary endpoints. The significance threshold for secondary endpoints was 0.05. Forty (40) subjects were initially randomized in order to ensure that 30 subjects completed all test sessions.

Data management and statistical analyses were performed by Celerion (Lincoln, NE). Statistical summarizations and comparisons were calculated using SAS version 9.3 (SAS, Cary, NC). Phoenix WinNonlin version 6.3 (Certara, Princeton, NJ) was used to calculate non-compartmental nicotine pharmacokinetics and subjective measure response parameters.

In all analyses, Vuse Solo was compared to CC and nicotine gum, and no comparisons were made between the two comparator products. Mixed effects models for analysis of variance were used to compare product liking, intent to use again, and product effects. Sequence, period, and product were included as fixed effects and subject-nested-within-sequence was included as a random effect. A mixed model analysis of covariance was used to compare the urge to smoke parameters, with sequence, period, and product included as fixed effects, subject-nested-within-sequence as a random effect, and baseline score as a covariate. All subjective effects parameters were analyzed on the original scale.

Measured plasma nicotine concentrations that were below the lower limit of quantitation (0.200 ng/ml) were set to one-half of the lower limit of quantitation for data summarization and statistical analysis. Individual nicotine concentrations were adjusted for the presence of nicotine at baseline as described by Shiffman et al.^41^, and all pharmacokinetic parameters were calculated from the adjusted concentrations. A mixed-effects model was used to compare the nicotine uptake parameters.

Statistical comparisons of C_max_ and AUCs were conducted to see if geometric means were equal (i.e., geometric mean ratio = 1). AUCs and C_max_ were analyzed on the natural log scale. Sequence, period, and investigational product were included as fixed effects and subject-nested-within-sequence was included as a random effect. Wilcoxon signed rank test was used in T_max_ comparisons. Any subjects with T_max_ ≥ 120 min were considered not to have used the study products effectively during the test session, and the subjective effects and PK data analyses were performed both with and without the potentially compromised test session data. The data presented herein excludes those subjects. The statistical comparisons with all subjects are provided in the supplemental materials.

The maximum increase in systolic and diastolic blood pressures and heart rate were compared using a mixed-effects model. If there was no increase from baseline (prior to product use) and the difference was zero or less, zero was reported for the maximum increase.

## Results

The original randomized, controlled, open-label crossover study was designed assess elements of AL of three closed system ENDS relative to CC and nicotine gum. No comparisons between ENDS products were made, and only the data for Vuse Solo G2 and the high- and low-AL comparators are reported in this manuscript. Data for the other two products relative to the comparator products will be reported in a separate publication.

### Study population

Of the 93 subjects screened, 40 were randomized and 38 (95%) completed the three test sessions for CC, nicotine gum, and Vuse Solo. One subject was withdrawn from the study by the investigator following a blood draw-related vasovagal reaction before administration of study product in the first test session. Another voluntarily withdrew from participation at the beginning of the second test session due to discomfort with the repeated blood draws. Of those that were screened and not randomized, the majority failed requirements of inclusion/exclusion criteria (n = 40). The remainder either chose not to continue to enrollment or were lost to follow-up (n = 13).

Three subjects had nicotine T_max_ values ≥ 120 min, an indicator of ineffective or inappropriate product use. Examination of individual PK curves revealed that two subjects had a second rise in nicotine levels several hours after initial product use, suggesting use of another nicotine-containing product during the session. Another subject had an essentially flat curve with only a slight rise at two hours. Statistical comparisons outlined in the protocol assume ad libitum use of each product for a specified amount of time at the beginning of each test session, and subjective measures are intended to assess this experience. Where the PK profiles indicated additional product use or non-use, the test session data for those subjects were deemed non-representative of the product being tested and were excluded from the final analysis presented below.

The demographic and baseline characteristics of all randomized subjects are summarized in Supplementary Table 1. The study population was predominantly male (70%), white (95%), and the majority were non-Hispanic (95%) with a mean age of 41.2 (standard deviation [SD] = 11.01) years. Mean individual cigarette use was 18 cigarettes per day (SD = 4.26) and the mean smoking duration was 20 years (SD = 12.7). All subjects were self-reported smokers of CC with limited ENDS experience. The level of cigarette dependence at screening was moderate based on Fagerström Test for Nicotine Dependence scores (mean total score 5.5 [SD = 1.54])^42^.

### Product use

Pre-to-post-use product weight differences for Vuse Solo were assessed following the prescribed 10 min of ad libitum product use for the relevant test session to confirm product use. The mean e-liquid weight difference, and thus the amount of e-liquid used, was 0.034 ± 0.022 g (range 0.005–0.107 g). This amount of e-liquid corresponds to approximately 1.63 mg of nicotine.

### Subjective measures

Statistical comparisons were made between Vuse Solo and each comparator. As illustrated in Table 1, use of Vuse Solo resulted in scores for product liking (area under the effect curve [AUEC_15-240_], E_max_, and overall product liking), and intent to use again (E_max_) that were consistently significantly lower (p < 0.0001) than those for CC and significantly higher than those for nicotine gum (p ≤ 0.0003). Positive effects scores after use of Vuse Solo were intermediary between smoking CC and use of nicotine gum (statistically significant differences [p ≤ 0.0224] for all comparisons). Conversely, negative effects scores for Vuse Solo were significantly lower as compared to nicotine gum (p ≤ 0.006 for both AUEC_15-240_ and E_max_) with no significant differences compared to scores for CC.

**Table 1:** Statistical comparisons of subjective measures parameters between Vuse Solo and the high- and low-AL comparators.

Parameter^a^	Vuse Solo (N = 37)	Usual brand cigarette (N = 38)	Nicotine gum (N = 36)
Product liking (AUEC_15-240_)	1245.18*^†^	1735.65	866.68
Product liking (E_max_)	6.61*^†^	8.83	5.04
Overall product liking	5.56*^†^	8.14	3.58
Overall intent to use again (E_max_)	4.19*^†^	8.98	2.24
Positive effects (AUEC_15-240_)	757.14*^†^	925.74	578.44
Positive effects (E_max_)	6.13*^†^	7.02	4.23
Negative effects (AUEC_15-240_)	356.32^†^	341.43	499.50
Negative effects (E_max_)	2.58^†^	2.91	4.32
Urge to smoke (AUEC_0-15_)	96.86*^†^	70.60	110.41
Urge to smoke (AUEC_0-240_)	1776.12*	1609.04	1861.75
Urge to smoke (E_min_)	5.01*	2.68	5.84
Urge to smoke (T_min_, minutes)	16.11^†^	14.66	33.04

The high- and low-AL comparators were not compared to each other.

*AUEC*_*15–240*_ area under the effect curve from 15 to 240 min after the start of product use, *E*_*max*_ maximum effect score, *AUEC*_*0–15*_ area under the effect curve from 0 to 15 min after the start of product use, *AUEC*_*0–240*_ area under the effect curve from 0 to 240 min after the start of product use, *E*_*min*_ minimum effect score, *T*_*min*_ time to minimum urge to smoke.

^a^Least squares means from mixed-effect models are presented.

*Significantly different from usual brand cigarette; p < 0.05.

^†^Significantly different from nicotine gum; p < 0.05.

**Figure 1:**
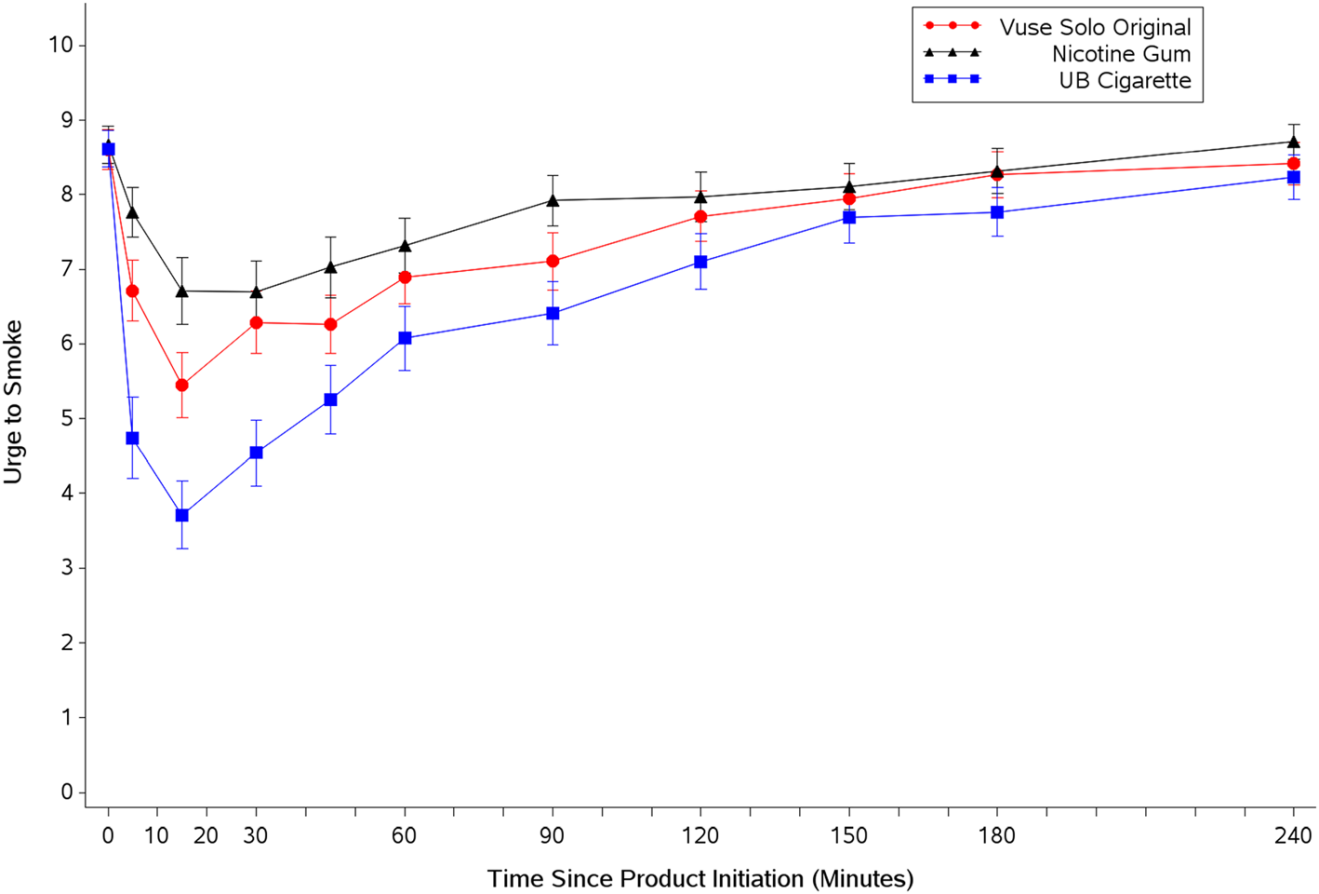
Arithmetic mean (SE) urge to smoke response profiles over four hours after initiation of product use. *SE* standard error, *UB* usual brand.

Mean urge to smoke scores (UTS) during the first 15 min following initiation of product use (AUEC_0-15_) were significantly higher for Vuse Solo than for CC (p < 0.0001) but significantly lower than for nicotine gum (p = 0.0164; Table 1). Over the entire 4-h test session (AUEC_0-240_), UTS scores for Vuse Solo were significantly higher than for CC (p = 0.0080) and equivalent to those for nicotine gum. Table 1 and Fig. 1 indicate that UTS scores decreased more slowly and to a lesser extent after using Vuse Solo as compared to smoking, but more quickly and to a greater extent when compared to nicotine gum. By 150 min after product use, UTS scores for all products had converged and by 240 min they had returned to near-baseline values (Fig. 1).

The differences in degree of effect (AUEC_0-15_, AUEC_0-240_ and E_min_) for UTS scores between use of Vuse Solo and CC were significant (p ≤ 0.0001, 0.0080 and < 0.0001, respectively). Although Vuse Solo did not provide the same magnitude of smoking relief as CC, the time to minimum UTS (T_min_) was not significantly different between Vuse Solo and CC (16.1 and 14.7 min, respectively; Table 1). This contrasts with the T_min_ comparison between Vuse Solo and nicotine gum, as use of Vuse Solo provided significantly greater relief of smoking urges during the first 15 min after the start of product use (p = 0.0164) and reached the minimum urge significantly faster (p = 0.0110) (Table 1).

### Nicotine Pharmacokinetics

**Figure 2:**
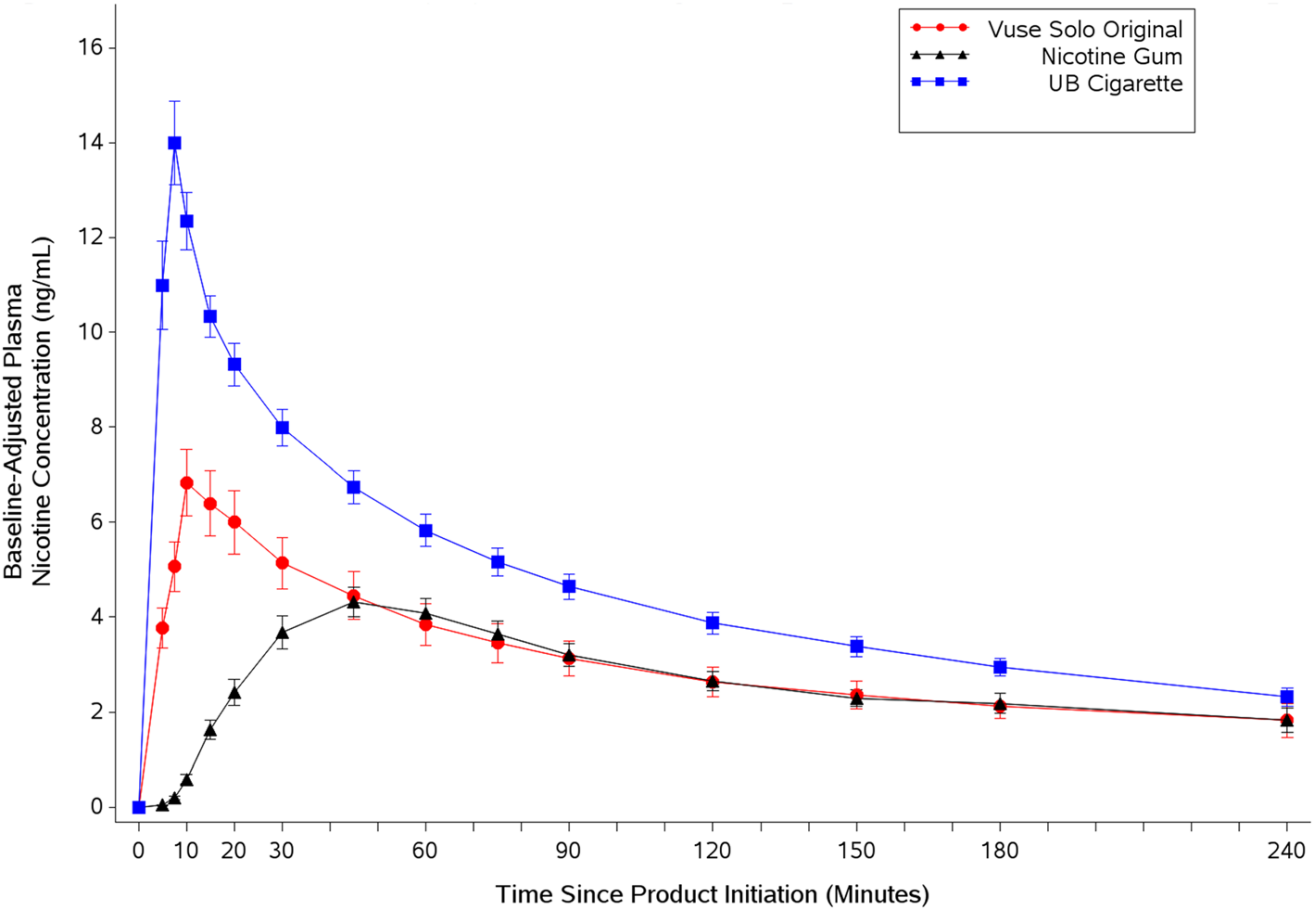
Arithmetic mean (SE) baseline-adjusted plasma nicotine concentration profiles. *SE* standard error, *UB* usual brand.

As illustrated in Fig. 2, plasma nicotine concentrations increased rapidly within 15 min of the start of CC and Vuse Solo use and more gradually after initiation of nicotine gum use, peaking at approximately 45 min. By 240 min, mean plasma nicotine levels had declined to approximately 2 ng/ml for all conditions. Statistical comparisons of baseline-adjusted nicotine PK parameters are summarized in Table 2. Geometric least square means were used for C_max_ and AUC statistical comparisons; median values were used for T_max_ comparisons. Mean nicotine uptake during the first 15 min following the start of product use (AUC_nic 0–15_) was significantly lower (p < 0.0001) with use of Vuse Solo than with CC but was significantly higher (p < 0.0001) than with use of nicotine gum. Over the four-hour sampling period, the mean total nicotine uptake (AUC_nic 0–240_) after use of Vuse Solo was significantly lower (p < 0.0001) than after smoking but not significantly different (p = 0.9029) than with use of nicotine gum. Further, the mean C_max_ was significantly lower with use of Vuse Solo than with CC (5.48 and 14.07 ng/ml, respectively; p < 0.0001) but was significantly higher than with use of nicotine gum (3.99 ng/ml; p = 0.0096). The median time to reach the maximum nicotine concentration (T_max_) with use of Vuse Solo was slightly but significantly longer (p < 0.0001) than with use of CC, likely due in part to the CC being fully consumed before the end of the 10-min product use window; T_max_ for Vuse Solo was significantly shorter (p < 0.0001) than for nicotine gum (Table 2).

**Table 2:** Statistical comparisons of baseline-adjusted plasma nicotine uptake parameters between Vuse Solo and the high- and low-AL comparators.

PK parameter^a^	Vuse Solo G2 (N = 37)	Usual brand cigarette (N = 39)	Nicotine gum (N = 36)
C_max_ (ng/ml)	5.48*^†^	14.07	3.99
AUC_nic 0–15_ (ng*min/ml)	46.83*^†^	140.7	4.67
AUC_nic 0–240_ (ng*min/ml)	557.2*	1082	550.3
T_max_ (minutes)	10.13*^†^	7.62	45.03

The high- and low-AL comparators were not compared to each other.

*C*_*max*_ maximum concentration, *AUC*_*nic* 0–15_ area under the curve from 0 to 15 min after the start of product use, *AUC*_*nic* 0–240_ area under the curve from 0 to 240 min after the start of product use, *NA* not assessed, *T*_*max*_ time to maximum concentration.

^a^Geometric least square means were used for the C_max_ and AUC statistical comparisons; median values were used for T_max_ statistical comparisons. Arithmetic means for C_max_ were 7.3, 15.0, and 4.6 ng/ml, respectively, for Vuse Solo G2, UB, and nicotine gum.

*Significantly different from usual brand cigarette; p < 0.05.

^†^Significantly different from nicotine gum; p < 0.05.

### Physiologic effects

Mean systolic and diastolic blood pressure and pulse rates at baseline before the start of use of all study products were similar, ranging from a mean of 109.8 to 110.3 mmHg, 68.6 to 69.4 mmHg, and 68.4 to 70.9 bpm, respectively. No statistically significant differences (p > 0.05) were seen between any products in maximum increase in systolic blood pressure (8.13, 8.62, and 8.34 mmHg with Vuse Solo, CC, and nicotine gum, respectively), diastolic blood pressure (5.93, 5.62 and 7.02 mmHg), or heart rate (9.90, 12.20 and 9.08 bpm).

### Adverse events

All study products were well tolerated under the conditions of use during the study. At total of 54 AEs, all rated as mild, occurred during the Vuse Solo, CC, and nicotine gum study periods reported in this manuscript (see Supplemental Table S2). Eight AEs were reported in 6 subjects (16%) during the Vuse Solo study period, 12 AEs in 7 subjects (18%) during the CC study period, and 34 AEs in 15 subjects (38%) during the nicotine gum study period. In this study, none of the reported AEs were judged by the PI to be related or possibly related to use of Vuse Solo because they did not follow a reasonable temporal sequence from use of the IP or could be reasonably explained by other factors. The AEs judged to be related or possibly related to nicotine gum use were abdominal distension (1), dizziness (3), dyspepsia (1), feeling hot (1), headache (3), hiccups (1), increased lymphocyte count (1), throat irritation (1), oral discomfort (1), and nausea (2). The AEs judged to be related or possibly related to cigarette use were dizziness (1), headache (1), and nausea (1). No serious adverse events were reported.

## Discussion

This study was designed to compare elements of AL for Vuse Solo G2 to high- (CC) and low- (nicotine gum) AL comparators using a framework generally similar to that proposed by the FDA CTP^8^. After a single ad libitum use of Vuse Solo, regular smokers scored Vuse Solo as intermediary between CC and NRT gum for measures of product liking, overall intent to use again, and positive effects. They reported fewer negative effects after use of Vuse Solo and CC than NRT gum, and use of Vuse Solo repressed urges to smoke in the same time frame as did smoking. This data, combined with the results from other published studies including Vuse Solo (both G1 and G2)^22,23,35^, support the conclusion that the AL of Vuse Solo is less than that of a cigarette but higher than that of NRT.

The other published studies on Vuse Solo include two AL studies on Vuse Solo G1 in tobacco and menthol flavors^22,23^ and an AL study of JUUL in which Vuse Solo (likely G2) was included as a comparator^35^. The 29 mg per cartridge e-liquid in the Stiles et al. studies correspond to 4.8% nicotine by weight (~ 57 mg/ml) e-liquid in this study, and the design and functionality of all components are identical across products but for the minor changes in the G1 and G2 cartridge. The C_max_ and AUC_0-15_ values reported in Stiles et al. (2017: 4.67 ng/ml and 42.64 ng*min/ml) are generally similar to those obtained in this study (5.48 ng/ml and 46.83 ng*min/ml). Likewise, Goldenson et al.^44^ reported a C_max_ for Vuse Solo of 6.8 ng/ml after a standardized 10-puff use. The C_max_ and AUC_0-60_ values for Vuse Solo were similar to or below those of the JUUL products. A range of C_max_ values has been reported in other published studies of Vuse Solo. For example, Hong et al. (2022) report a mean C_max_ of 6.91 ng/ml (Vuse Solo G2) after 10-min ad libitum product use among a population of exclusive smokers and dual users of cigarettes and ENDS^39^. In another study, a small sample of experienced ENDS users achieved relatively high nicotine uptake after five minutes of ad libitum Vuse Solo use (mean C_max_ of 13.6 ng/ml)^11^. The overall conclusions regarding AL of Vuse Solo and other ENDS are consistent: their AL falls between that of the high- and low-AL comparators.

Consistent with other published literature on ENDS^9,20,43,44^, Vuse Solo was able to reduce early smoking urges to a significantly greater degree than nicotine gum, and time to maximum reduction of urges after Vuse Solo use was closer to that of smoking. Since acute nicotine cravings have been shown to lead to smoking lapse within 10 min^45^, any replacement product for CC must deliver nicotine at a sufficient rate and level so as to adequately satiate the smoker and prompt switching^46^. The pharmacokinetic results suggest that Vuse Solo can serve as an appropriate replacement product for CC among adult smokers accustomed to a fairly rapid nicotine uptake and thus a faster onset of craving relief and reductions in urge to smoke.

Further, nicotine abuse liability is highly dependent on the form of the product and the mechanism of nicotine uptake, and the speed of delivery to the central nervous system (slower onset, less reinforcing)^47^. With use of smokeless tobacco and NRT, absorption via the buccal route is slower and the products are less reinforcing^48,49^. Since ENDS products and cigarettes both create aerosols and both deliver nicotine to the brain with the same rapidity^50^, a product such as Vuse Solo may support product switching from CC.

Subjective effects in general, and product liking scores in particular, are also considered important in evaluating AL of tobacco products and are a standard component of many clinical investigations of AL. While the absolute product liking scores for ENDS products are not directly comparable across studies, the interpretation of product liking or similar subjective measures are consistent. Scores for CC are highest, followed by Vuse Solo, with oral NRT products substantially lower. Such results hold true in other studies of ENDS, despite differences in product design and variability in individual nicotine uptake^44,51,52^. Furthermore, across all studies of Vuse Solo, the observed differences in product liking scores between the Vuse Solo products (Vuse Solo G1 [Original, Menthol] and Vuse Solo G2 [Original, Menthol, and other flavors]) did not translate to substantial differences in nicotine PK parameters during acute ad libitum use^22,23,39^.

The confinement design employed in this study successfully minimized the drop-out rates that were noted in the previous ambulatory studies conducted by Stiles and colleagues^22,23^ Clinical confinement also ensured some product familiarization prior to each test session. Due to the profile of the PK curve and the known timeframe around nicotine elimination, the data collection period was reduced to 240 min from 360 min in the previous Stiles et al. studies^22,23^ This reduced the sampling burden on the subjects. Subjects tended to use less e-liquid during the 10-min test sessions in the present study compared to the previous study with Vuse Solo G1 (Original) (0.034 vs. 0.048 g), but the mean nicotine C_max_ and AUC_nic 0–15_ were comparable^22^. The median time to maximum concentration (T_max_) after use of Vuse Solo G2 occurred slightly sooner (in the current study than in the previous study (10.1 vs. 15.2 min)^22^. One potential explanation for these differences across the studies could be increased familiarity with the product use with the addition of scheduled product acclimation sessions during confinement. Other research with exclusive smokers has reported relatively low nicotine uptake with initial use of ENDS and increased uptake following experience with the product^53–56^.

There are several limitations of the present study design. The focus of this study was to compare elements of AL between Vuse Solo and high- and low-AL comparators and to assess how adult smokers generally unfamiliar with ENDS experienced Vuse Solo during initial use. The exclusion of experienced ENDS users, dual users of ENDS and CCs, and non-users of tobacco products could limit the generalizability of these findings to consumers of different categories of tobacco products. However, exclusive smoking remains the most prevalent tobacco use behavior^57^ and Vuse Solo is intended to be a product for smokers who choose to continue using nicotine-containing products but who want to switch product use to one that is lower on the risk continuum.

Another potential limitation of the study design was the fact that subjects used the Vuse Solo product ad libitum during the first 10 min of the test session. Although a prescribed puffing regimen would have reduced variability in the nicotine uptake, self-selection of puffing behaviors provides ecological validity^58^. Thus, ad libitum use was included so that the measures of AL would more closely reflect actual product use by smokers. A ten-minute “unit of use” for ENDS products was previously used by RAIS in similar studies (Stiles et al. 2017; Stiles et al. 2018) and is not uncommon in the research literature (e.g., Harvanko et al. 2020, Yingst et al. 2019)^22,23,59,60^. Therefore, the same 10-min time frame of Vuse Solo product use was employed in this study and in Hong et al. (Part 2 in this series) to allow for comparison across studies^39^. Lastly, because the commercial Vuse Solo e-liquids are manufactured with only one nicotine concentration, only one concentration was assessed in this study. As such, the impact of different nicotine concentrations on AL measures was not evaluated. The previous studies with Vuse Solo G1 (Original and Menthol at three nicotine concentrations) showed that the AL for all the Vuse Solo products, regardless of nicotine concentration, was between that of the comparators^22^.

Notably, there were fewer AEs during the Vuse Solo study periods than during the use of either comparator product. This is consistent with a previous AL study using Vuse Solo G1^22^. In the present study, no AEs were judged to be related to Vuse product use. One explanation for this may be the limited time with which subjects interacted with the Vuse product (seven 10-min uses) versus nicotine gum (seven 30-min uses) and their usual brand cigarette (multiple periods of ad libitum use). However, in the companion PK study with similar product use parameters, two AEs (oropharyngeal pain and gastroesophageal reflux) were identified as related or possibly related to Vuse Solo use^39^. Furthermore, AEs related to Vuse Solo use in short-term switching studies have been reported and commonly include headache, nausea, and cough^38,40^. Reports of higher number of AEs during use of NRT gum relative to other products are not uncommon in studies with exclusive smokers, which may suggest that they are not familiar with sensations of oral product use^22^. Increased AEs during the NRT gum test sessions could also result from prolonged withdrawal effects after overnight abstinence. In general, the numbers of AEs reported in clinical research of vapor products such as Vuse Solo are generally low and characterized overall as mostly mild, transient, and expected^61^.

In summary, the findings of the current study are consistent with those observed in our previous evaluation of Vuse Solo with the first-generation cartridge design. Both studies formed part of the evidence submitted to the FDA requesting marketing authorization and support the conclusion that the AL for Vuse Solo (4.8% nicotine, Original flavor) is lower than that for CC but higher than that for nicotine gum. The lower AL of Vuse Solo compared to CC is consistent with accumulating evidence for the overall ENDS category^35,41,56,62^. In addition, the nicotine PK profile within the first 15 min of Vuse Solo use was much closer to that of CC use than to nicotine gum use and suggests the potential for alleviation of smoking urges and cravings and increases the likelihood of product adoption^45^. Changes in product experience over time may influence nicotine delivery and uptake with ENDS, and other characteristics may need to be considered to optimize satisfaction of the experience for smokers to completely switch from CC.

## Supplementary Information

Supplementary Information.

## Data Availability

The applicable data generated or analyzed during this study are included in this manuscript (and its supplementary tables). Additional datasets generated and/or analyzed during the study are available from the corresponding author on reasonable request.
