# Supplementary material for: Part one: abuse liability of Vuse Solo (G2) electronic nicotine delivery system relative to combustible cigarettes and nicotine gum
Source: Sci Rep. 2022 Dec 21;12:22080. doi: 10.1038/s41598-022-26417-2 (PMC9772348; doi:10.1038/s41598-022-26417-2)
Supplement: Supplementary file 1 — Supplementary Information. [file 41598_2022_26417_MOESM1_ESM.docx]

# Part One: Abuse Liability of Vuse Solo Relative to Combustible Cigarettes and Nicotine Gum Supplementary Material

Supplementary Figure S1. Study Schematic


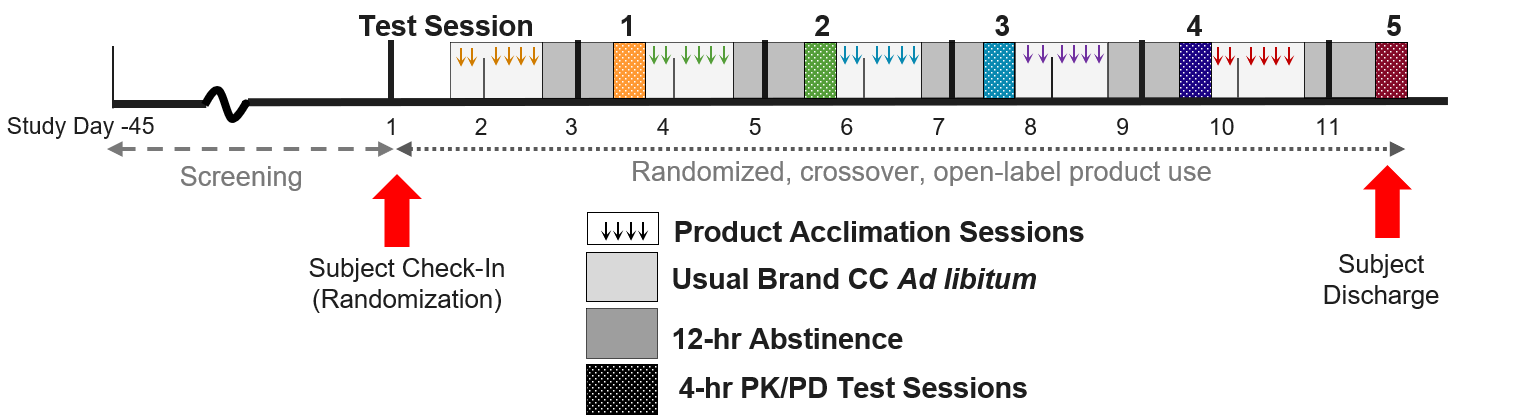


Supplementary Table S1. Demographic and baseline characteristics

| Parameter | All Enrolled Subjects (n=40) |
| --- | --- |
| Age (years) | 41.2 (±11.01) |
| Weight (kg) | 84.1 (±17.44) |
| Height (cm) | 172.9 (±8.74) |
| BMI (kg/m^2^) | 28.07 (±5.040) |
| Sex |  |
| Male | 28 (70%) |
| Female | 12 (30%) |
| Ethnicity |  |
| Hispanic/Latino | 2 (5%) |
| Not Hispanic/Latino | 38 (95%) |
| Race |  |
| White | 38 (95%) |
| Black/African American | 1 (3%) |
| Native Hawaiian/Other Pacific Islander | 0 (0%) |
| Asian | 0 (0%) |
| American Indian/Alaska Native | 1 (3%) |
| Multiple | 0 (0%) |
| Smoking |  |
| Number of years smoked | 19.7 (±12.7) |
| Number of cigarettes smoked per day | 18.1 (±4.26) |
| Level of dependence on nicotine (FTND) | 5.5 (±1.54) |

Data are mean (±SD) or number (%). Abbreviations: BMI = body-mass index, FTND = Fagerström Test for Nicotine Dependence

Supplementary Table S2. Summary of Adverse Events

| **Product (number of subjects reporting AEs / number of episodes)** | **Total Number of AEs Judged as ‘Not Related’ or ‘Unlikely Related’^a^** | **Total Number of AEs Judged as ‘Related’ or ‘Possibly Related’^b^** |
| --- | --- | --- |
| **Usual Brand Cigarette (7 / 12)** | **9** | **3** |
| Contusion | 1 | 0 |
| Dizziness | 0 | 1 |
| Headache | 0 | 1 |
| Hyperhidrosis | 1 | 0 |
| Lymphadenopathy | 1 | 0 |
| Nausea | 0 | 1 |
| Pain in extremity | 1 | 0 |
| Presyncope | 1 | 0 |
| Vessel puncture site pain | 2 | 0 |
| Vessel puncture site reaction | 1 | 0 |
| Vessel puncture site swelling | 1 | 0 |
| **Vuse Solo (6 / 8)** | **8** | **0** |
| Headache | 2 | 0 |
| Muscle spasms | 1 | 0 |
| Paranasal sinus discomfort | 1 | 0 |
| Presyncope | 1 | 0 |
| Sinus congestion | 1 | 0 |
| Sinus headache | 1 | 0 |
| Vessel puncture site pain | 1 | 0 |
| **Nicotine Gum (5 / 34)** | **19** | **15** |
| Abdominal distension | 0 | 1 |
| Arthralgia | 1 | 0 |
| Back pain | 2 | 0 |
| Blood urine present | 1 | 0 |
| Burning sensation | 1 | 0 |
| Constipation | 3 | 0 |
| Diarrhoea | 1 | 0 |
| Dizziness | 0 | 3 |
| Dyspepsia | 0 | 1 |
| Erythema | 1 | 0 |
| Feeling hot | 0 | 1 |
| Headache | 0 | 3 |
| Hiccups | 0 | 1 |
| Lymphocyte count increased | 2 | 1 |
| Nausea | 0 | 2 |
| Oral discomfort | 0 | 1 |
| Paraesthesia | 1 | 0 |
| Peripheral coldness | 1 | 0 |
| Presyncope | 2 | 0 |
| Throat irritation | 0 | 1 |
| Vessel puncture site pain | 2 | 0 |
| White blood cell count increased | 1 | 0 |
| During each 48-hour study period, subjects used the Study IP to which they were randomly assigned during Product Acclimation Sessions and a Test Session. All AEs were assigned to the randomized Study IP and relatedness to use of that study product was assessed by the Principal Investigator (PI) responsible for study conduct.  ^a^ AEs that were judged as ‘not related’ or ‘unlikely related’ were those that did not follow a reasonable temporal sequence from use of the IP or could be reasonably explained by other factors (i.e., underlying disease, complications, or concomitant drugs).  ^b^ AEs that were judged as ‘related’ or ‘possibly related’ were those that followed a reasonable temporal sequence from use of the IP (including the course after withdrawal of the IP) and that can be excluded as being possibly caused by other factors. | | |

Supplementary Table S3. Statistical comparisons of subjective measures parameters between Vuse Solo and the high- and low-AL comparators with data from all subjects

| Parameter ^a^ | Vuse Solo  (G2) | Usual brand cigarette | Nicotine gum |
| --- | --- | --- | --- |
|  | (N=38) | (N=39) | (N=38) |
| Product liking (AUEC_15-240_) | 1237.80*^†^ | 1735.99 | 890.52 |
| Product liking (E_max_) | 6.61*^†^ | 8.83 | 5.15 |
| Overall product liking | 5.56*^†^ | 8.14 | 3.58 |
| Overall intent to use again (E_max_) | 4.19*^†^ | 9.0 | 2.24 |
| Positive effects (AUEC_15-240_) | 747.77*^†^ | 926.02 | 579.66 |
| Positive effects (E_max_)^b^ | **6.26^†^** | 7.02 | 4.34 |
| Negative effects (AUEC_15-240_) | 344.83^†^ | 341.64 | 506.53 |
| Negative effects (E_max_) | 2.57^†^ | 2.91 | 4.27 |
| Urge to smoke (AUEC_0-15_) | 96.37*^†^ | 70.60 | 110.29 |
| Urge to smoke (AUEC_0-240_) | 1771.06* | 1609.04 | 1856.71 |
| Urge to smoke (E_min_)^b^ | **4.92*^†^** | 2.68 | 5.86 |
| Urge to smoke (T_min_, minutes) | 16.32^†^ | 14.66 | 32.00 |
| ^a^ Least squares means from mixed-effect models are presented  ^b^ There were changes in statistical significance for these parameters when all subjects were included in the analysis versus the analysis presented in the body of the manuscript.  * Significantly different from usual brand cigarette; p < 0.05  ^†^ Significantly different from nicotine gum; p < 0.05  Note: The high- and low-AL comparators were not compared to each other  Abbreviations: AUEC_15–240_, area under the effect curve from 15 to 240 minutes after the start of product use; E_max_, maximum effect score; AUEC_0–15_, area under the effect curve from 0 to 15 minutes after the start of product use; AUEC_0–240_, area under the effect curve from 0 to 240 minutes after the start of product use; E_min_, minimum effect score; T_min_, time to minimum urge to smoke. | | | |

Supplementary Table S4. Statistical comparisons of baseline-adjusted plasma nicotine uptake parameters between Vuse Solo and the high- and low-AL comparators with data from all subjects

| PK Parameter^a^ | Vuse Solo  (G2) | Usual brand cigarette | Nicotine  gum |
| --- | --- | --- | --- |
|  | (N=38) | (N=39) | (N=38) |
| C_max_ (ng/mL) | 5.61*^†^ | 14.06 | 4.11 |
| AUC_nic 0–15_ (ng*min/mL) | 49.44*^†^ | 140.8 | 4.61 |
| AUC_nic 0–240_ (ng*min/mL) | 570.8* | 1082 | 558.8 |
| T_max_ (minutes) | 10.14*^†^ | 7.62 | 45.03 |
| ^a^ Geometric least square means were used for the C_max_ and AUC statistical comparisons; median values were used for T_max_ statistical comparisons  * Significantly different from usual brand cigarette; p<0.05.  ^†^ Significantly different from nicotine gum; p<0.05.  Note: The high- and low-AL comparators were not compared to each other  Abbreviations: C_max_, maximum concentration; AUC_nic 0–15_, area under the curve from 0 to 15 minutes after the start of product use; AUC_nic 0–240_, area under the curve from 0 to 240 minutes after the start of product use; NA, not assessed, T_max_, time to maximum concentration. | | | |
